# Developmental Change in Associations Between Mental Health and Academic Ability Across Grades in Adolescence: Evidence from IRT-Based Vertical Scaling

**DOI:** 10.3390/bs16010078

**Published:** 2026-01-06

**Authors:** Yuanqiu Ma, Youyou Duan, Yunxiao Qi, Ying Hu, Tour Liu

**Affiliations:** 1 Faculty of Psychology, Tianjin Normal University, Tianjin 300387, China; myqee23@163.com (Y.M.); qyx20000602@163.com (Y.Q.); 2 Key Research Base of Humanities and Social Sciences of the Ministry of Education, Academy of Psychology and Behavior, Tianjin Normal University, Tianjin 300387, China; 3 Tianjin Social Science Laboratory of Students’ Mental Development and Learning, Tianjin 300387, China

**Keywords:** vocabulary ability, item response theory, vertical scaling, internalizing symptoms, adolescence

## Abstract

Adolescence is a critical period when rapid cognitive maturation coincides with heightened emotional vulnerability. This study examined the dynamic association between academic ability and mental health across early adolescence, focusing on vocabulary ability as a core indicator of academic ability. Using large-scale data from Grades 1–12 (N = 13,412), a vertically scaled vocabulary ability scale was constructed based on Item Response Theory (IRT) and the Non-Equivalent Anchor Test (NEAT) design to achieve cross-grade comparability. Fixed-parameter calibration was then applied to an independent cross-sectional sample of middle school students (Grades 7–9, N = 401) in Tianjin, combined with the DASS-21 to assess internalizing symptoms (depression, anxiety, stress). Hierarchical multiple regression analyses revealed that higher vocabulary ability was significantly associated with lower levels of depression, anxiety, and stress, with the negative association strongest in Grade 8. The present study provides new empirical evidence for understanding the interactive mechanisms between academic and psychological development during adolescence. Methodologically, the study demonstrates the value of IRT-based vertical scaling in establishing developmentally interpretable metrics for educational and psychological assessment.

## 1. Introduction

Early adolescence is a developmental window in which rapid cognitive gains co-occur with heightened emotional vulnerability. During this period, neuroplasticity is markedly enhanced, facilitating learning and growth in academic ability; at the same time, emotion-regulatory functions of the prefrontal cortex are not yet fully mature, rendering adolescents more susceptible to mood lability and mental health problems (Casey et al., 2019; Pfeifer & Allen, 2021). Globally, mental health problems among adolescents constitute a major public health concern, with recent estimates indicating that approximately 10–20% experience anxiety or depressive symptoms and that prevalence rates have continued to rise in recent years (Lu et al., 2024; World Health Organization, 2025). In China, systematic reviews and meta-analyses similarly report high prevalence rates of internalizing symptoms among children and adolescents, suggesting a substantial mental health burden comparable to, or in some estimates exceeding, global averages (Z. Chen et al., 2023; Zhou et al., 2024).

Within China’s Confucian-heritage educational system, academic achievement occupies a central social and cultural position and is widely regarded as a primary pathway to social mobility and family honor. This emphasis, reinforced by parental expectations and social comparison, often translates into sustained academic pressure during adolescence, increasing vulnerability to internalizing problems such as depression and anxiety. At the same time, students in Chinese participating regions consistently demonstrate strong academic performance relative to international benchmarks (Organisation for Economic Co-operation and Development, 2023). Comparative research suggests that this coexistence of high achievement and high academic pressure is particularly salient in East Asian education systems, giving rise to the widely discussed “high achievement–high pressure” profile (Steare et al., 2023). In response, China’s Ministry of Education issued the Double Reduction policy in 2021, aiming simultaneously to reduce academic burden and strengthen school-based mental health services. Against this backdrop, examining the coordinated development of academic ability and mental health in early adolescence is of immediate practical importance and of policy relevance for advancing educational equity and improving school mental-health support systems.

Adolescence is widely recognized as a critical developmental period characterized by elevated risk for mental health problems. A large-scale meta-analysis of 192 epidemiological studies identified the peak age of onset for most mental disorders at approximately 14.5 years, with a substantial proportion of first-episode symptoms emerging before age 18 (Solmi et al., 2022). In Western populations, clear sex differences have been documented, with boys showing higher rates of externalizing problems and girls exhibiting greater vulnerability to internalizing symptoms such as anxiety and depression (Salk et al., 2017). In the Chinese context, cultural socialization processes—including collectivist norms and high societal and academic expectations—may further predispose adolescents to internalized psychological difficulties (X. Chen et al., 2013). Recent systematic reviews and large-scale studies indicate that approximately 22–26% of Chinese children and adolescents report depressive symptoms, and about one quarter report anxiety symptoms, placing China at a medium-to-high level internationally (Xu et al., 2024). Longitudinal and repeated cross-sectional evidence further suggests that internalizing symptoms increase steadily during the junior-high years (roughly ages 12–15), particularly among girls and adolescents exposed to high academic pressure (Liu et al., 2024; Y. Sun et al., 2023; Wu et al., 2022). Thus, this body of evidence highlights early adolescence as a critical window for mental-health risk emergence and an important target period for educational and preventive interventions.

Academic ability is a dynamic construct that evolves with cognitive maturation and educational experience. Longitudinal and growth-model research demonstrates that the development of core academic skills is shaped by the interplay of educational environment, socioeconomic resources, and neurocognitive maturation, showing nonlinear trajectories characterized by alternating acceleration and plateau phases (Erbeli et al., 2021; Little et al., 2021). In reading, fluency growth is fastest in the early grades but slows thereafter, and students exhibit heterogeneous developmental profiles rather than a single path (Khanolainen et al., 2024). The reciprocal association between reading and mathematics is stronger in elementary school but tends to weaken or change direction in secondary education (Gnambs & Lockl, 2023). Longitudinal evidence from Chinese children similarly reveals persistent divergence in vocabulary growth (van der Kleij et al., 2023). With the onset of early adolescence (approximately 10–14 years), neural and cognitive systems undergo major reorganisation: prefrontal functions and executive control develop rapidly, and abstract and formal-operational reasoning begin to emerge (Best & Miller, 2010). Such ability differences are socially manifested through grades, standardized tests, and classroom ranking, triggering social-comparison effects that may intensify achievement anxiety and self-efficacy disparities, leading some students to difficulties in academic pressure and emotional regulation (Jiang et al., 2021). Conversely, higher language and vocabulary ability facilitate emotional expression and social communication, thereby reducing psychological distress (Hentges et al., 2021; Mellado, 2025). Academic ability and mental health are believed to exert bidirectional influences. According to the Attentional Control Theory, anxiety disrupts goal-directed attention and consumes working-memory resources, impairing task performance (Eysenck et al., 2007). Excessive stress and anxiety heighten attention to threat cues, reduce working-memory efficiency, and decrease task persistence, whereas chronic anxiety or depression is associated with lower classroom engagement and achievement (Owens et al., 2012; Linnenbrink-Garcia & Pekrun, 2014).

Cross-sectional studies have consistently revealed a negative association between mental-health indicators and academic ability (Steinmayr et al., 2016); however, such evidence only captures static relationships at a single time point. To compare the strength of this association across developmental stages and to identify its trajectory or lagged effects, longitudinal designs are required (Pekrun et al., 2022). Cross-study comparisons indicate that most longitudinal research supports a negative relationship between mental health and academic ability, yet the magnitude and direction of this association vary across samples, statistical models, and control variables. For instance, longitudinal tracking studies in European samples have found that depressive symptoms predict subsequent declines in academic performance; however, when baseline ability and socioeconomic background are statistically controlled, the association often weakens or becomes non-significant (López-López et al., 2021; Wickersham et al., 2023). In Chinese adolescent samples, some studies have reported a persistent negative predictive effect of anxiety on subsequent Chinese-language achievement, whereas others suggest that this effect may be limited to short-term fluctuations or may vary depending on the measurement approach employed. These inconsistencies not only reflect the stage-specific and context-dependent nature of the relationship between mental health and academic ability but also highlight notable limitations in current psychometric practices (W. Chen, 2025; Ye et al., 2019). At present, many longitudinal studies of academic development still rely on the Classical Test Theory (CTT) framework, in which measurement typically depends on within-grade standard scores, raw scores, or teacher ratings. Such scores primarily reflect an individual’s relative position within a specific sample or test form, rather than representing an interval-scaled latent ability; consequently, cross-grade comparisons may inflate or underestimate true growth (Protopapas et al., 2014). Moreover, item difficulty, scoring standards, and content coverage often differ substantially across grades or test versions. Without employing scaling or linking techniques to place all forms on a common scale, it is impossible to ensure measurement invariance and conceptual equivalence of the latent construct (Kolen & Brennan, 2014). Therefore, longitudinal or cross-sectional studies based solely on CTT scores may conflate true developmental change with measurement error, hindering accurate identification of stage-specific features in the link between mental health and academic ability (Gorter et al., 2015).

Overall, developmental evidence concerning the relationship between academic ability and mental health during early adolescence (approximately 12–15 years) in the Chinese context remains limited. Existing studies have primarily focused on cross-sectional samples or a single educational stage, offering little insight into developmental continuity. To address this gap, the present study adopts a developmental perspective that integrates educational measurement and mental health research to examine whether the association between academic ability and internalizing symptoms follows a dynamic, stage-specific pattern during early adolescence. Vocabulary comprehension was selected as a core indicator of academic ability, given its foundational role in language understanding and its relevance across academic domains (Ricketts et al., 2020). Methodologically, Item Response Theory (IRT) and vertical scaling were used to construct a unified developmental vocabulary ability scale spanning Grades 1–12. This scale was then applied to an independent junior-high sample (Grades 7–9) using a fixed-item-parameter calibration procedure (König et al., 2021), enabling developmentally comparable ability estimates. This measurement framework allowed us to examine cross-grade variation in the association between academic ability and internalizing symptoms without confounding developmental differences with measurement artifacts.

Based on developmental theory and prior empirical evidence, the present study tested two primary hypotheses. First, vocabulary ability was expected to be negatively associated with internalizing symptoms during early adolescence, such that adolescents with higher vocabulary ability would report lower levels of depression, anxiety, and stress (H1). Second, the strength of this association was hypothesized to vary across grade levels, reflecting developmental differences in academic demands and emotional vulnerability during early adolescence (H2). In addition, by comparing IRT-based vertically scaled vocabulary ability estimates with within-grade standardized raw scores, the study examined whether conclusions about academic–mental health associations depend on the measurement framework used.

## 2. Materials and Methods

### 2.1. Participants

This study comprised two rounds of data collection. Dataset 1, used for the standardization and scaling of academic achievement measures, was drawn from eight public schools located in Shenzhen, a major city in southern China. The dataset covered primary, junior, and senior high school levels. A multi-site convenience sampling design was adopted, with intact classrooms serving as the testing units. A total of 13,536 native Mandarin-speaking students participated in the assessment. Data screening excluded any cases meeting one or more of the following criteria: (1) missing responses exceeding 10%; and (2) aberrant response patterns, such as invariant answers across items or near-random response behavior. After data cleaning, the final valid sample comprised 13,412 students, aged 6 to 18 years, encompassing Grades 1 through 12 within China’s compulsory-education and general high-school system. Table 1 presents the sample size and descriptive statistics of raw scores by grade level.

Dataset 2 focused on junior-high students and was used to examine the association between academic ability and mental health during early adolescence. Participants were drawn from four public junior high schools in Tianjin. After data cleaning, cases with missing responses or aberrant answering patterns were removed, resulting in a final valid sample of 401 students. Among them, 48.6% were female, and 43.0% were only children. The final sample was distributed across Grade 7 (*n* = 131; *M* = 12.36 years, SD = 0.51), Grade 8 (*n* = 132; *M* = 13.40 years, SD = 0.52), and Grade 9 (*n* = 138; *M* = 14.30 years, SD = 0.50).

### 2.2. Measures

#### 2.2.1. Depression Anxiety Stress Scales-21 (DASS-21)

The Chinese version of the DASS-21 was administered to assess adolescents’ internalizing symptoms (Gong et al., 2010; Lovibond & Lovibond, 1995). The DASS-21 is a self-report questionnaire consisting of 21 items that measure negative emotional states experienced during the previous week. It comprises three subscales—Depression, Anxiety, and Stress—each containing seven items. All items are rated on a 4-point Likert scale ranging from 0 = “Did not apply to me at all” to 3 = “Applied to me very much or most of the time.” Subscale scores are obtained by summing responses across the seven items within each domain, with higher scores indicating greater symptom severity. In the present sample, all three subscales demonstrated good internal consistency reliability, with Cronbach’s α = 0.83 for Depression, α = 0.85 for Anxiety, and α = 0.86 for Stress.

#### 2.2.2. Standardized Vocabulary Comprehension Tests

Students’ vocabulary ability was assessed using the Chinese Vocabulary Comprehension Test for Primary and Secondary Students. The theoretical framework and original item bank were developed based on the pioneering IRT scaling research of Cao (1999). The item bank consists of 649 five-option multiple-choice items, derived from the high-frequency and core vocabulary of Chinese language textbooks for Grades 1–12, encompassing major semantic categories (nouns, verbs, adjectives, and function words) specified in the national curriculum standards. Each item presents the target word within a short contextual sentence, and students choose the most appropriate meaning from five alternatives. All items were dichotomously scored (1 = correct, 0 = incorrect). To enable vertical linking across grades, the test employed a Non-Equivalent Groups Anchor Test (NEAT) design, in which each grade-level form contained common anchor items as well as grade-specific items. The proportion of anchor items ranged from 18% to 52%. Results from multiple pilot and large-scale administrations confirmed the psychometric robustness of the test. Internal-consistency coefficients were high across grade-level forms (Cronbach’s α = 0.86–0.88), and the vertical-scaling outcomes were stable and reliable. Details of the anchor-item distribution are reported in Table 2.

### 2.3. Procedure

The present study collected two complementary datasets to address different but interrelated research objectives. Dataset 1 was collected to support IRT calibration and vertical scale construction across Grades 1–12, whereas Dataset 2 was used to examine the association between vocabulary ability and mental health during early adolescence (Grades 7–9). To obtain stable item-parameter estimates, IRT calibration and vertical scaling typically require large samples at each grade level (Embretson & Reise, 2000). Accordingly, Dataset 1 included 13,536 students, with approximately one thousand participants per grade, providing a sufficient empirical basis for cross-grade parameter estimation and linking. For the regression analyses, Dataset 2 comprised 401 junior-high students, which is consistent with commonly accepted guidelines for detecting small-to-moderate effects, including interaction terms, in multiple regression models (Cohen, 1992).

Data for both datasets were collected from primary and secondary school students in two economically developed regions in China, in close collaboration with participating schools. Assessments were administered at the classroom level during regular school hours. Prior to testing, trained research assistants provided standardized instructions to all participants, emphasizing that participation was entirely voluntary and that responses would be kept strictly confidential. For Dataset 1, vocabulary assessments were administered by trained teachers or research staff at participating schools following a standardized testing protocol. The assessment typically required approximately 20–30 min to complete. For Dataset 2, both the vocabulary test and the mental health questionnaire (DASS-21) were administered by trained research assistants in cooperation with school staff. All participants completed the assessments individually within a single session lasting approximately 30–45 min. Data collection for both datasets was completed in late September 2023.

All study procedures were conducted in accordance with established ethical standards. Written informed consent was obtained from all participants and their parents or legal guardians prior to data collection. Participation was voluntary, data were anonymized and securely stored, and participants were informed of their right to withdraw at any time. If a participant’s questionnaire responses indicated potential emotional distress, the research team informed the school in accordance with pre-established collaboration procedures, allowing school staff to provide appropriate follow-up support.

### 2.4. Vocabulary Ability Scale Construction

To enable meaningful comparisons of vocabulary ability across grades, the scale construction process followed three core steps. First, IRT was applied to estimate latent vocabulary ability while accounting for item difficulty and discrimination. Second, because different grade-specific test forms were used, a vertical linking procedure was implemented to place all item parameters onto a common metric, ensuring cross-grade comparability. Third, based on the linked item parameters, a fixed-parameter calibration approach was used to obtain comparable ability estimates for the junior high school sample analyzed in subsequent models. Together, these procedures ensured that observed grade differences in vocabulary ability reflected developmental variation rather than measurement artifacts.

#### 2.4.1. IRT Modeling

The present study employed IRT to estimate students’ latent vocabulary ability and to construct a common scale suitable for cross-grade comparisons. Unlike raw test scores, which are inherently sample and test-form-dependent, IRT-based ability estimates provide a model-based metric that supports meaningful comparisons across different test forms and grade levels (Embretson & Reise, 2000; Kolen & Brennan, 2014). Several IRT models were compared in terms of model fit and parameter stability (see Appendix A.1), and the two-parameter logistic (2PL) model was selected as the unified framework for vertical scaling due to its balance between interpretability and stability.

Under the 2PL model, the probability that student *j* with latent ability $\theta_{j}$ correctly answers item *i* was defined as:

(1) $${P(U}_{ij}\;=\;1|\theta_{j},a_{i},b_{i})=\;\frac{1}{1+e^{-a_{i}(\theta_{j}-b_{i})}},$$

where $a_{i}$ is the discrimination parameter (sensitivity to differences in ability) and $b_{i}$ is the difficulty parameter, the ability level at which the probability of a correct response is 0.50 (Baker & Kim, 2004).

#### 2.4.2. Vertical Linking

Because vocabulary tests administered at different grade levels necessarily differ in item composition and difficulty, vertical linking is required to ensure that latent ability estimates are expressed on a common developmental scale rather than grade-specific metrics (Lord, 1980; Kolen & Brennan, 2014). The study employed a NEAT design to link scales across grade levels. Two principal strategies were considered for placing item parameters from different grades on a common scale: concurrent calibration and separate calibration (Kolen & Brennan, 2014). The concurrent-calibration approach assumes that a single IRT model provides an adequate fit across grades; however, given the wide developmental range of the present dataset (Grades 1–12), this assumption was considered restrictive. (Embretson & Reise, 2000). To enhance flexibility and accommodate grade-specific characteristics, we adopted a separate-calibration strategy: item parameters were first estimated independently within each grade, followed by post hoc linking across grade-level scales.

Under separate calibration, two independently estimated scales that share common anchor items can be related through an approximately linear transformation (Lord, 1980; Kolen & Brennan, 2014). The transformation from scale *p* to the reference scale can be expressed as:

(2) $$\theta^{\left(\mathrm{ref}\right)}=A_{p}\times \theta^{\left(p\right)}+B_{p},$$

where *A_p_* > 0 (slope) and *B_p_* (intercept) are linking constants. For the 2PL model, item parameters transform as:

(3) $$a_{i}^{\left(ref\right)}=\frac{a_{i}^{\left(p\right)}}{A_{p}},$$

(4) $$b_{i}^{\left(ref\right)}=\;A_{p}\;b_{i}^{\left(p\right)}+B_{p}$$

Constants were estimated by minimizing discrepancies between transformed anchor-item functions across the two scales (Hambleton et al., 1991).

**Figure 1:**
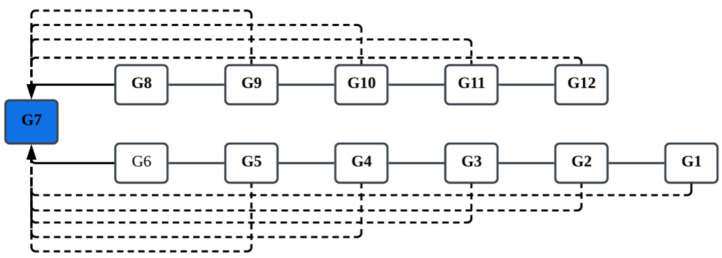
Schematic of the vertical linking pathway. *Note*. The blue band denotes the G7 reference scale. Solid lines: chained linking paths; dashed lines: direct links; arrows indicate the direction of transformation.

The study implemented chained linking to vertically connect scales across Grades 1–12, using Grade 7 (G7) as the reference scale. All subsequent transformations and linkages were ultimately anchored to the G7 metric. Grade 7 was selected as the reference level because it lies near the midpoint of the K–12 developmental continuum, thereby minimizing the propagation and accumulation of linking errors across multiple transformations (Battauz, 2015). As shown in Figure 1, these bidirectional linking paths extend both forward and backward across adjacent grades, forming an integrated scale network that unifies all test forms into a single continuous measurement continuum.

The implementation process consisted of three sequential phases: (1) Each grade-level dataset was independently calibrated under the 2PL model to obtain 12 initial sets of item parameters. (2) Using an anchor set (excluding items exhibiting significant differential item functioning), the chained-linking procedure was applied to estimate the final transformation constants *A_p_* and *B_p_* from each grade to the G7 reference scale. (3) All grade-level item parameters were transformed to the G7 scale according to Equations (2) and (3), thereby establishing a unified cross-grade measurement scale.

### 2.5. Statistical Analysis

All statistical analyses were conducted in R version 4.2.3 (R Core Team, 2023). For each grade-level dataset, the 2PL model was fitted using the Expectation–Maximization (EM) algorithm implemented in the mirt package (Chalmers, 2012). Prior to parameter estimation, essential unidimensionality was evaluated by fitting a unidimensional IRT model and inspecting the M_2_-based global fit indices. Adequate fit (RMSEA < 0.08, CFI > 0.90, TLI > 0.90; Hu & Bentler, 1999) was taken as evidence supporting essential unidimensionality. To stabilize the linking and ensure anchor-item invariance, all designated anchor items were screened through Differential Item Functioning (DIF) analyses using likelihood-ratio tests for nested IRT models (Thissen et al., 1993). False Discovery Rate (FDR) correction (Benjamini & Hochberg, 1995) was applied to control for multiplicity (α = 0.05), and graphical inspection of item difficulty (*b*) parameters was used to further exclude biased anchors, ensuring cross-grade stability.

To determine the optimal linking function, four commonly used linking methods available in the equateIRT package (Battauz, 2015) were compared: Haebara, Stocking–Lord, Mean–Mean, and Mean–Sigma. The comparison was based on two criteria: (*a*) smoothness and monotonic separation of Test Characteristic Curves (TCCs) across grades, and (*b*) standard errors of the linking constants, where smaller errors indicate greater stability (Kolen & Brennan, 2014). After identifying the optimal method, fixed-parameter calibration was conducted to rescore all examinees, and ability estimates (*θ*) were obtained using the Expected A Posteriori (EAP) method in mirt, allowing visualization of grade-level latent ability trajectories. To examine how the development of vocabulary ability moderates its association with adolescents’ internalizing symptoms, the fixed-parameter calibration approach was applied to the cross-sectional junior high school dataset (Dataset 2). Using the linked item parameters from Dataset 1 as fixed references, response data from Dataset 2 were analyzed in the mirtCAT package (Chalmers, 2025) to obtain comparable latent ability estimates for Grades 7–9 directly on the established Grade 7 reference scale. These IRT-based vocabulary ability estimates served as the independent variable in subsequent analyses.

The three subscales of psychological health—Stress, Depression, and Anxiety—served as dependent variables. Relationships among variables were examined using hierarchical multiple regression analysis. In the first step, gender and only-child status were entered as control variables. as prior meta-analyses had demonstrated consistent gender differences in internalizing symptoms (Salk et al., 2017), and only-child status has been shown to have cultural specificity in the Chinese sociocultural context (Lin et al., 2021). In the second step, grade level (with Grade 7 as the reference group) and IRT-based vocabulary ability were entered to test their main effects. In the third step, an interaction term (Vocabulary Ability × Grade) was added to test whether the association between vocabulary ability and internalizing symptoms varied across developmental stages. All continuous predictors were mean-centered to reduce multicollinearity and facilitate the interpretation of main effects. Model explanatory power was assessed using the change in *R*^2^ (Δ*R*^2^) (Cohen et al., 2003).

## 3. Results

### 3.1. Construction and Verification of the Vertical Scale

**Figure A1:**
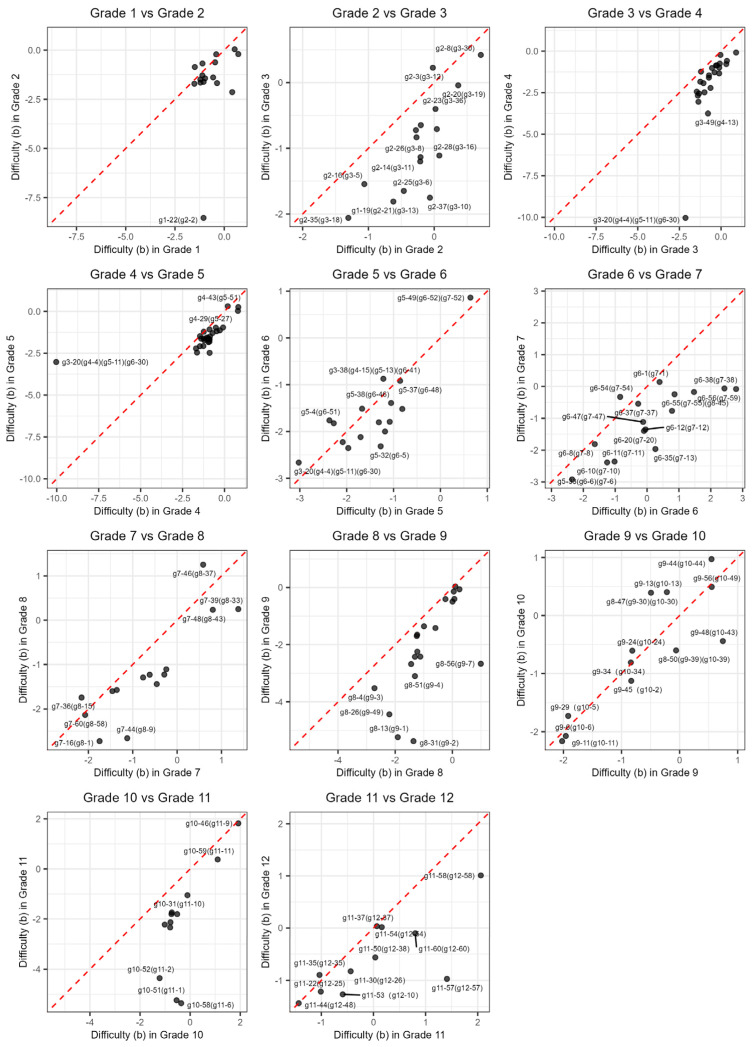
Stability of anchor item difficulty (*b*) parameters across adjacent grades.

To ensure the stability of the linking, DIF was examined using IRT Likelihood Ratio Tests (LRT) combined with visual inspection of scatterplots of item difficulty parameters (*b*) between adjacent grades (see Appendix A.2, Figure A1). After screening, six items with irregular response patterns were removed, and thirteen items exhibiting severe DIF were downgraded to non-anchor status. The final anchor proportions for each grade are reported in Table 3.

Independent 2PL model estimation for each grade (Table 3) showed satisfactory model fit across test forms: most exhibited CFI/TLI values above 0.90 and RMSEA values below 0.05, supporting essential unidimensionality. A few higher-grade forms (e.g., G10: CFI = 0.86, TLI = 0.85, RMSEA = 0.05) demonstrated slightly weaker fit, yet the overall fit remained acceptable for vertical scale construction.

**Figure 2:**
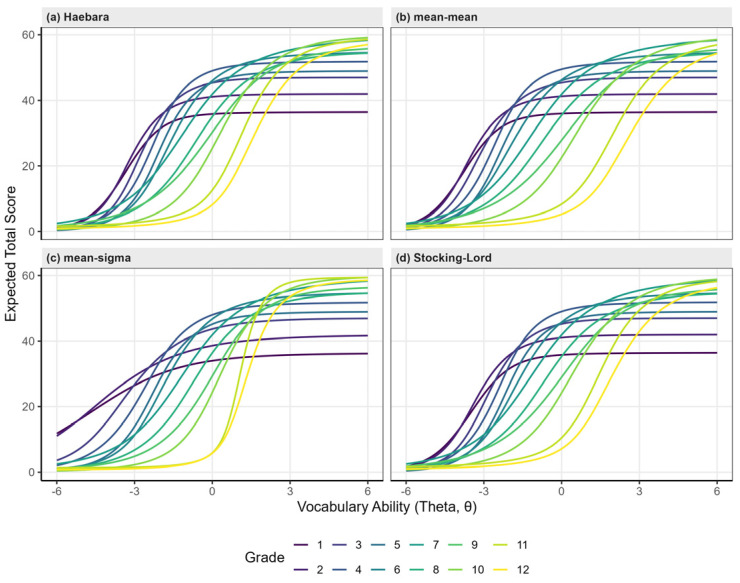
Test Characteristic Curve (TCC) Clusters Generated by Four Linking Methods.

To establish a stable vertical scaling system across Grades 1–12, four IRT-based linking methods (Haebara, Stocking–Lord, Mean–Mean, and Mean–Sigma) were compared (see Appendix B, Table A2). Using the equated parameters, Test Characteristic Curves (TCCs) were plotted for each grade (Figure 2).

The Test Characteristic Curves (TCCs) for each grade exhibited the typical S-shaped growth pattern, where expected scores increased with higher levels of latent ability (*θ*). Moreover, as grade level increased, the curves shifted progressively rightward, indicating that students in higher grades required greater latent ability (*θ*) to achieve comparable expected scores. This systematic rightward shift reflects increasing test difficulty across grades, providing evidence for the vertical validity of the scale. Among the four linking methods, both the Haebara and Stocking–Lord approaches yielded the smoothest and most orderly progression of TCCs while maintaining stable linking constants with smaller standard errors. Considering both model stability and transformation precision, the Haebara method was selected as the final procedure for vertical scale construction.

**Figure 3:**
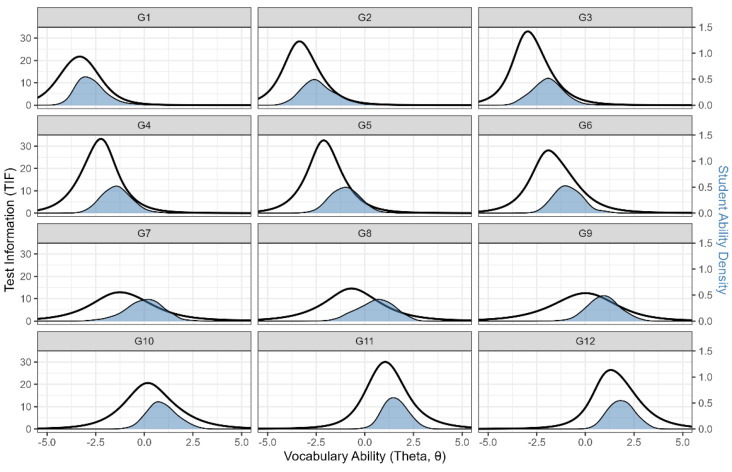
Test Information Functions (TIFs) and Ability Distributions Across Grades. *Note.* Solid lines represent the TIFs, and the blue curves represent the estimated ability distributions of the examinees across grades.

Consequently, the descriptive statistics of the scaled ability and item parameters are summarized in Appendix B (Table A3). In the final longitudinal scale, item discrimination parameters were generally high (mean = 1.34, *SD* = 0.58), and grade-level mean item difficulty parameters (*b*) ranged from –3.21 to 1.75, encompassing both lower and higher regions of the latent ability continuum. Figure 3 presents a comparison between the Test Information Functions (TIFs) and the estimated ability distributions across grades. The TIF reflects the measurement precision at each level of latent ability, where higher peaks indicate greater precision (Lord, 1980). Alignment between the TIF peaks and the centers of the ability distributions indicates that measurement precision is maximized at the ability levels most densely represented in the sample, resulting in lower measurement error for the target population. Across all twelve grades, the peaks of the TIFs closely aligned with the centers of the respective ability distributions, indicating that each grade-level test achieved optimal precision for its target population (Embretson & Reise, 2000). These findings suggest that the constructed longitudinal scale demonstrates high measurement reliability and structural stability across grades.

### 3.2. Developmental Trajectory and Group Variability

**Figure 4:**
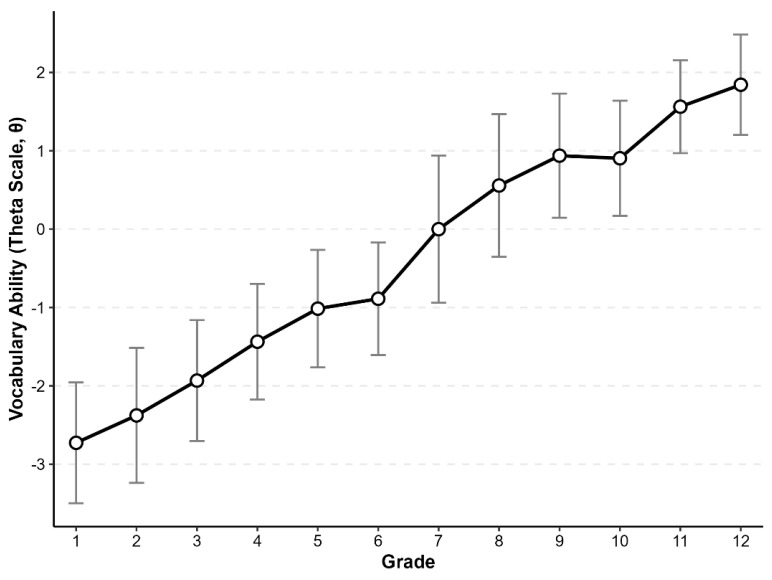
Developmental Trends of Vocabulary Ability Across Grades.

As shown in Figure 4, estimates of students’ latent ability (*θ*) from Dataset 1 (Shenzhen sample) across Grades 1–12 illustrate the developmental trend and inter-group variability in vocabulary ability. With increasing grade level, vocabulary ability exhibited a nonlinear and stage-like developmental pattern. The Grades 6–9 period showed the steepest growth slope, indicating this stage as a critical period of accelerated ability development. The standard deviation of ability scores expanded progressively from the primary grades, suggesting widening individual differences; these differences peaked during Grades 7–9 and then gradually narrowed in the higher grades.

**Figure 5:**
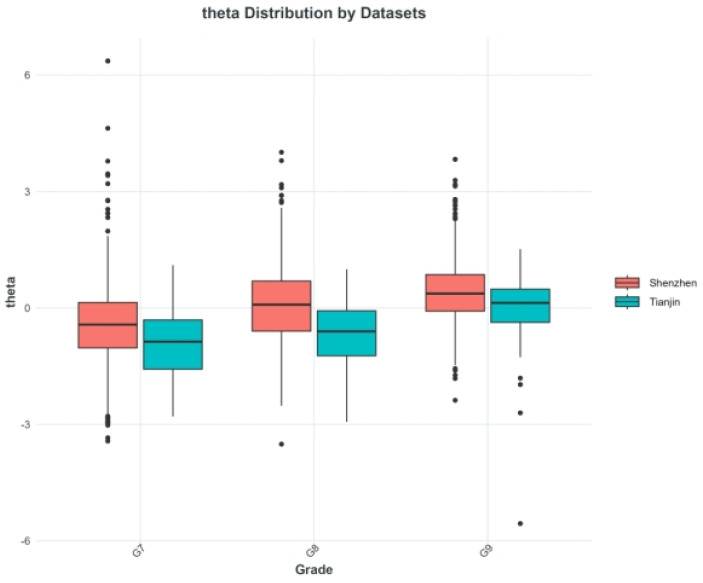
Distribution of standardized latent ability (*θ*) across Grades 7–9 for students from Shenzhen (Dataset 1) and Tianjin (Dataset 2).

Boxplots were used to visualize the distribution of students standardized latent ability (*θ*) in Grades 7–9 from Shenzhen (Dataset 1) and Tianjin (Dataset 2), as shown in Figure 5. The comparison revealed a parallel growth trend in ability levels across grades for both regions, indicating a generally consistent developmental pattern. Meanwhile, students from Shenzhen consistently demonstrated higher latent ability levels than those from Tianjin, suggesting systematic regional differences in vocabulary ability performance.

### 3.3. Descriptive Statistics and Bivariate Correlations

The core analyses focused on the junior secondary school sample from Tianjin, with descriptive and correlational statistics presented in Table 4. Significant positive intercorrelations were observed among the three internalizing symptom dimensions (depression, anxiety, and stress), suggesting strong covariation among these negative emotional states. Vocabulary comprehension reflects a developmental progression across grades: students’ vocabulary ability increased significantly with grade level, with a strong positive correlation observed between grade and ability (*r* = 0.57, *p* < 0.001). Moreover, Vocabulary ability showed statistically significant negative correlations with both depression (*r* = −0.14, *p* = 0.006) and anxiety (*r* = −0.16, *p* = 0.002), while its negative association with stress did not reach significance (*r* = −0.10, *p* = 0.051).These results preliminarily suggest that higher vocabulary ability is associated with lower levels of depression and anxiety symptoms.

### 3.4. Cross-Grade Association Between Vocabulary Development and Internalizing Symptoms

Table 5 presents the results of hierarchical multiple regression analyses predicting three internalizing dimensions—depression, anxiety, and stress. Unstandardized regression coefficients (*B*) are reported, with vocabulary ability operationalized as IRT-derived latent scores estimated on a common vertical scale. The analyses examined the predictive effects of grade, vocabulary ability, and their interaction, while controlling for gender and only-child status.

In Model 1, which included only control variables (gender and only-child status), the explained variance was low (R^2^ ≈ 0.01), and the overall F-test was non-significant. In Model 2, grade (with seventh grade as the reference group) and vocabulary ability (*θ*) were added, resulting in a significant improvement in explanatory power (ΔR^2^ ≈ 0.04–0.05). Compared with seventh graders, eighth graders reported higher levels of depressive, anxiety, and stress symptoms, with statistical significance varying across outcomes (Table 5). Ninth graders also showed higher levels of depressive and stress symptoms relative to seventh graders, whereas differences in anxiety were not statistically significant. Across all three internalizing dimensions, vocabulary ability demonstrated a significant negative predictive effect, indicating that higher vocabulary ability was associated with lower levels of depression (*B* = −1.28), anxiety (*B* = −1.04), and stress (*B* = −1.08).

**Figure 6:**
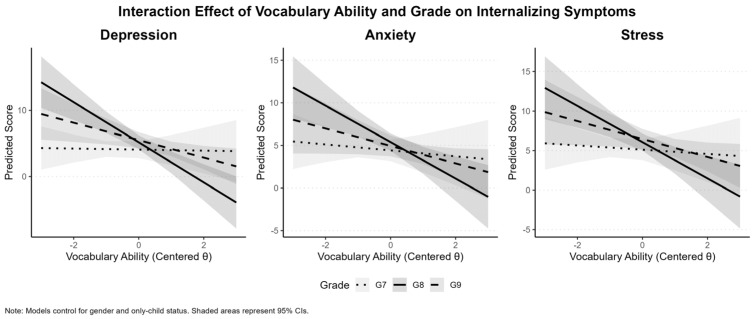
Interaction Effects of Vocabulary Ability × Grade.

When the interaction term between vocabulary ability and grade was added in Model 3, the model’s explanatory power showed a modest improvement (ΔR^2^ ≈ 0.01), indicating a small but statistically significant contribution of the interaction terms. The interaction between vocabulary ability and grade was significant for eighth grade across all three outcomes. Specifically, the vocabulary ability × eighth grade interaction was negatively associated with depression (*B* = −2.58), anxiety (*B* = −1.56), and stress (*B* = −1.77). In contrast, the vocabulary ability × ninth grade interaction did not reach statistical significance for any of the outcomes. As illustrated in Figure 6, the negative association between vocabulary ability and internalizing symptoms was more pronounced in Grade 8 than in Grade 7, reflecting steeper negative slopes as indicated by the larger negative interaction coefficients in Model 3, with a consistent directional pattern across the three internalizing dimensions.

To evaluate the robustness of the measurement approach, a comparative analysis using within-grade standardized raw scores was conducted (Appendix C, Table A4). Consistent with the main analyses, the IRT-based latent ability scores demonstrated comparatively stronger predictive validity and greater developmental sensitivity than conventional raw-score indicators.

## 4. Discussion

### 4.1. Overview of Main Findings

This study examined the association between academic ability and mental health during early adolescence from a developmental perspective. To enable cross-grade comparability, we used IRT to construct a vertically linked vocabulary scale spanning Grades 1–12, thereby placing ability estimates on a common developmental metric. Using this common metric, we examined associations between vocabulary ability and three internalizing symptoms and tested whether these associations varied across Grades 7–9. The results indicated that higher vocabulary ability was associated with lower levels of depression, anxiety, and stress. After controlling for gender and only-child status, these associations remained statistically significant, and the Grade 8 interaction terms suggested a relatively steeper negative association compared with Grade 7, albeit with modest incremental variance explained. Overall, the present study provides empirical evidence on the developmental interplay between academic and psychological functioning in adolescence and illustrates the utility of IRT-based vertical scaling for cross-grade measurement in the Chinese context. These findings contribute empirical evidence to research on academic–mental health co-development in adolescence and demonstrate how IRT-based vertical scaling enables the identification of developmental patterns in academic–psychological associations across grades in the Chinese context.

### 4.2. Developmental Interpretation of Grade Differences

In a sample of junior secondary students, higher vocabulary ability was significantly associated with lower levels of depression, anxiety, and stress. This finding is consistent with longitudinal and review studies showing that children with weaker language skills are more likely to exhibit internalizing problems during late childhood and early adolescence (Bornstein et al., 2013; Hentges et al., 2021). Language ability may reduce internalizing symptoms through two primary pathways: by facilitating emotion regulation (e.g., cognitive reappraisal or linguistic distancing; Nook et al., 2020, 2025) and by enhancing social competence (e.g., improved peer interaction and emotional support; Wieczorek et al., 2024). In this context, stronger language ability may reflect richer emotional vocabulary and inner speech resources, which could support emotion labeling and cognitive reappraisal as well as more effective social communication. Consequently, students with higher vocabulary ability may report lower levels of internalizing symptoms. Furthermore, the negative association appeared relatively more pronounced in Grade 8 than in Grade 7. Within the Chinese middle-school curriculum structure, Grade 8 marks a significant increase in course difficulty and academic demands, which may heighten the relevance of language-related resources for emotional regulation and coping (J. Sun, 2024). In this context, the association between vocabulary ability and internalizing symptoms may become more salient during Grade 8, even if the overall effect size remains modest.

By contrast, although Grade 9 is generally associated with increasing pressure related to the high-stakes entrance examination, data collection in the present study took place at the beginning of the fall semester, prior to the peak period of exam-related stress. Prior research suggests that exam-related stress and emotional distress tend to intensify as high-stakes examinations approach rather than remaining constant across the school year, with peaks in mental health symptoms often observed during examination periods (George, 2024). As a result, the psychological burden typically associated with imminent entrance examinations may not yet have fully manifested at the time of assessment in the present study. Moreover, in the Chinese context, higher academic burden and examination-related pressure have been consistently associated with depressive and anxiety symptoms among adolescents (Wang et al., 2025). In addition, Grade 9 students may enter a more structured phase of exam preparation (e.g., standardized instruction and collective training), which could reduce between-student variability in study routines and perceived stress early in the semester. Such contextual arrangements may reduce between-student variability in emotional and stress responses during the early semester, thereby attenuating the statistical detectability of interaction effects at this stage.

### 4.3. Methodological Implications

Methodologically, this study goes beyond prior work that has relied primarily on CTT-based raw scores or within-grade standardized scores by adopting an IRT-based vertical scaling framework to support cross-grade comparability of vocabulary ability. By placing academic performance on a common latent scale rather than treating it as grade-specific or sample-dependent, this approach enabled developmentally interpretable comparisons across grades. This measurement strategy proved critical for identifying stage-specific patterns in the association between academic ability and internalizing symptoms. Using a unified ability scale, we were able to examine how the strength of academic–psychological associations vary across developmental stages, revealing a more pronounced association in early secondary school (especially Grade 8). Such patterns may be difficult to detect using conventional within-grade standardization, which removes between-grade variance and may obscure developmental differences.

The use of IRT calibration and linking aligns with established practices in large-scale assessments such as NAEP, PISA, and TIMSS, where common metrics are used to ensure comparability across forms, grades, and populations (Yamamoto & Mazzeo, 1992). Consistent with prior large-scale studies of academic skill development, the resulting vocabulary trajectory showed a nonlinear, stage-specific pattern, characterized by steady growth in primary school, accelerated growth with widening individual differences in early secondary school, and a leveling-off in upper secondary school (Peng et al., 2019). Importantly, by integrating a developmentally comparable academic ability scale with mental health outcomes, the present study illustrates how vertically scaled academic measures can advance research on the dynamic interplay between academic and psychological development during adolescence.

### 4.4. Limitations and Future Directions

Naturally, this study has several limitations. First, participants were recruited via convenience sampling from public schools in two economically developed Chinese cities, which may limit generalizability to other regions and school contexts. Neither dataset collected individual-level socioeconomic indicators or ethnicity; contextual information was limited to the city/school level. Because Dataset 1 primarily served IRT calibration and vertical scaling, we prioritized large grade-level samples and response-quality screening over detailed background variables, and Dataset 2 included only basic demographics for the regression models. Preliminary checks suggested possible DIF in a small number of items, and mean ability estimates differed across cities, supporting the need for broader multi-site calibration and validation. Future studies should sample more diverse regions and school types and collect richer contextual data to test invariance/DIF and improve the robustness and generalizability of the scale. Second, because the present study adopted a cross-sectional design, causal direction and true developmental trajectories could not be identified, and inferences about underlying mechanisms may be biased. In addition, academic ability was represented solely by vocabulary ability, excluding domains such as mathematics and reading comprehension, which limits a comprehensive understanding of academic–mental health covariation. Future research should employ multi-wave longitudinal designs to examine the temporal ordering and causal effects between vocabulary ability and internalizing symptoms. It is recommended that future work integrate mathematics and reading-comprehension measures within the unified scaling framework and include process-level tracking of mental health (e.g., emotion-regulation tasks or experience sampling) to enhance the explanatory power of mechanism testing. Furthermore, future studies could extend hybrid IRT approaches, such as multi-group IRT, Bayesian hierarchical modeling, and NEAT-linking comparisons across regions to systematically evaluate DIF and measurement fairness, thereby improving cross-regional comparability.

## 5. Conclusions

Using an IRT-based vertically scaled vocabulary metric, this study examined cross-grade patterns in the association between vocabulary ability and internalizing symptoms during early adolescence. Higher vocabulary ability was associated with lower levels of depression, anxiety, and stress, and this association showed modest but grade-specific variation, appearing relatively stronger in Grade 8 than in Grade 7. Beyond these substantive findings, the study highlights the value of vertically scaled measures: placing vocabulary ability on a common metric enabled the detection of stage-specific association patterns that may be attenuated or obscured when using within-grade standardized scores. Together, these results contribute evidence on academic–mental health interplay in early adolescence and underscore the importance of cross-grade comparable measurement for developmental research.

## Figures and Tables

**Table 1:** Descriptive statistics of the vocabulary ability test across Grades 1–12.

Grade	Sample	Mean Accuracy	Score SD
G1	1255	0.61	0.19
G2	1130	0.67	0.19
G3	1248	0.68	0.19
G4	1285	0.71	0.17
G5	1210	0.74	0.16
G6	1157	0.65	0.15
G7	1132	0.67	0.16
G8	1042	0.72	0.16
G9	925	0.7	0.13
G10	1032	0.63	0.16
G11	1002	0.62	0.15
G12	994	0.56	0.16

*Note*. Mean accuracy is the proportion correct relative to the total number of items on each grade-specific form.

**Table 2:** Structure of Grade-Level Test Forms (NEAT Design).

Grade	Total Items	Anchor Ratio	G1	G2	G3	G4	G5	G6	G7	G8	G9	G1	G11	G12	Cronbach’s α
G1	38	0.42	38												0.86
G2	44	0.66	16	44											0.87
G3	50	0.8	3	16	50										0.88
G4	54	0.87			24	54									0.88
G5	50	0.76			5	28	50								0.86
G6	56	0.54			2	6	16	56							0.88
G7	60	0.48					2	16	60						0.88
G8	57	0.54						1	14	57					0.88
G9	60	0.5							3	20	60				0.85
G10	60	0.4								2	12	60			0.88
G11	60	0.38										12	60		0.88
G12	60	0.18											11	60	0.89

*Note*. Anchor ratio = number of anchor items in the form ÷ total items.

**Table 3:** Model Fit Indices of the 2PL Model Across Grades.

Grade	Number of Items	Anchor Ratio (%)	CFI	TLI	RMSEA	Percentage of Fitted Items (%)
G1	37	40.5	0.97	0.97	0.03	83.8
G2	42	35.7	0.98	0.98	0.03	90.5
G3	47	46.8	0.99	0.99	0.02	91.5
G4	52	51.9	0.99	0.99	0.02	92.3
G5	49	30.6	0.99	0.99	0.01	95.9
G6	55	29.1	0.96	0.96	0.02	76.4
G7	60	26.7	0.93	0.93	0.03	75
G8	55	25.5	0.96	0.96	0.03	83.6
G9	58	31	0.93	0.93	0.03	93.1
G10	60	20	0.86	0.85	0.05	90
G11	60	20	0.9	0.89	0.04	86.7
G12	60	18.3	0.97	0.96	0.02	95

**Table 4:** Means, Standard Deviations, and Correlations.

	*M*	*SD*	1	2	3	4	5
1. Vocabulary ability	0.79	0.87	—				
2. Depression	4.52	5.07	−0.14 *	—			
3. Anxiety	4.91	4.93	−0.16 *	0.84 **	—		
4. Stress	5.69	5.15	−0.10	0.86 **	0.88 **	—	
5. Grade	2.01	0.82	0.57 **	0.06	−0.02	0.06	—

*Note*. Grade coding: 1 = G7; 2 = G8; 3 = G9; * *p* < 0.05, ** *p* < 0.01.

**Table 5:** Results of Hierarchical Multiple Regression Analyses Predicting Internalizing Symptoms.

Predictor	Depression			Anxiety			Stress		
	Model 1	Model 2	Model 3	Model 1	Model 2	Model 3	Model 1	Model 2	Model 3
	*B* (*SE*)	*B* (*SE*)	*B* (*SE*)	*B* (*SE*)	*B* (*SE*)	*B* (*SE*)	*B* (*SE*)	*B* (*SE*)	*B* (*SE*)
*Step 1: Control variables*									
Intercept	4.09 (0.42) *	3.24 (0.58) *	4.07 (0.66) *	4.79 (0.40) *	3.92 (0.56) *	4.42 (0.65) *	5.71 (0.42) *	4.53 (0.59) *	5.11 (0.68) *
Gender	0.46 (0.52)	0.45 (0.51)	0.54 (0.50)	0.94 (0.50)	1.11 (0.49) *	1.18 (0.49) *	0.90 (0.53)	1.01 (0.52)	1.07 (0.52) *
Only-child status	−0.82 (0.52)	−0.84 (0.51)	−0.96 (0.51)	−0.69 (0.50)	−0.88 (0.50)	−0.96 (0.50)	−0.82 (0.53)	−0.96 (0.53)	−1.05 (0.53) *
*Step 2: Main effects*									
Grade 8 vs. Grade 7		1.94 (0.65) *	1.06 (0.73)		1.50 (0.63) *	0.96 (0.71)		1.56 (0.67) *	0.94 (0.75)
Grade 9 vs. Grade 7		2.33 (0.73) *	1.41 (0.78)		1.12 (0.72)	0.52 (0.77)		1.98 (0.76) *	1.34 (0.81)
Vocabulary ability (θ)		−1.28 (0.31) *	−0.07 (0.57)		−1.04 (0.30) *	−0.30 (0.56)		−1.08 (0.31) *	−0.24 (0.58)
*Step 3: Interaction effects*									
Vocabulary ability × Grade 8			−2.58 (0.80) *			−1.56 (0.77) *			−1.77 (0.82) *
Vocabulary ability × Grade 9			−1.08 (0.73)			−0.59 (0.73)			−0.76 (0.76)
Model fit statistics									
R^2^	0.01	0.06	0.08	0.01	0.05	0.06	0.01	0.05	0.06
ΔR^2^		0.05 *	0.02		0.04 *	0.01		0.04 *	0.01
F	F (2, 383) = 2.01	F (5, 380) = 4.58 *	F (7, 378) = 4.87 *	F (2, 388) = 2.45	F (5, 385) = 4.27 *	F (7, 383) = 3.68 *	F (2, 387) = 2.37	F (5, 384) = 3.71 *	F (7, 382) = 3.35 *

*Note.* Unstandardized regression coefficients (*B*) and standard errors (*SE*) are reported. Vocabulary ability (*θ*) represents IRT-based latent ability scores estimated on a vertically linked common scale and was mean-centered prior to analysis. Gender was coded as 0 = male and 1 = female. Only-child status was coded as 0 = yes and 1 = no. Grade 7 served as the reference group. * *p* < 0.05.

## Data Availability

The data presented in this study are available on request from the corresponding author due to ethical restrictions.

## Appendix A. Model Comparison and Anchor Item Screening

### Appendix A.1. Model Comparison

To determine the most appropriate IRT model for calibration, three unidimensional models—the Rasch (1PL), two-parameter logistic (2PL), and three-parameter logistic (3PL)—were compared in terms of model fit (−2LL, AIC, BIC, RMSEA) and parameter stability across grades. As summarized in Table A1, the 2PL model achieved the best balance between fit and parsimony, whereas the 3PL model produced unstable guessing parameters in lower grades. Consequently, the 2PL model was selected as the unified framework for vertical scaling.

**Table A1:** Comparison of Model Fit Indices for Rasch, 2PL, and 3PL IRT Models Across Grades.

Grade	Model	−2LL	AIC	BIC	LRT vs. Simpler Model		
					χ^2^	df	*p*
G1	Rasch	54,058.24	54,136.25	54,336.51	-	-	-
	2PL	53,550.74	53,702.73	54,092.99	507.51	37	<0.001
	3PL	53,433.64	53,661.64	54,247.01	117.10	38	<0.001
G2	Rasch	53,284.42	53,374.43	53,600.78	-	-	-
	2PL	52,584.36	52,760.36	53,203.00	700.07	43	<0.001
	3PL	52,483.58	52,747.59	53,411.55	100.77	44	<0.001
G3	Rasch	65,058.92	65,160.93	65,422.52	-	-	-
	2PL	64,291.62	64,491.62	65,004.55	767.31	49	<0.001
	3PL	64,205.36	64,505.36	65,274.75	86.26	50	0.001
G4	Rasch	70,652.02	70,762.01	71,045.73	-	-	-
	2PL	69,670.86	69,886.85	70,443.97	981.16	53	<0.001
	3PL	69,613.00	69,937.01	70,772.69	57.84	54	0.335
G5	Rasch	57,829.68	57,931.68	58,191.70	-	-	-
	2PL	57,170.44	57,370.43	57,880.27	659.25	49	<0.001
	3PL	57,104.58	57,404.58	58,169.33	65.86	50	0.066
G6	Rasch	67,252.28	67,366.27	67,654.33	-	-	-
	2PL	66,310.58	66,534.58	67,100.58	941.70	55	<0.001
	3PL	66,072.40	66,408.40	67,257.40	238.18	56	<0.001
G7	Rasch	72,166.66	72,288.67	72,595.60	-	-	-
	2PL	71,096.06	71,336.05	71,939.86	1070.61	59	<0.001
	3PL	70,846.48	71,206.48	72,112.19	249.58	60	<0.001
G8	Rasch	58,128.10	58,244.10	58,531.14	-	-	-
	2PL	57,468.46	57,696.45	58,260.63	659.65	56	<0.001
	3PL	57,411.00	57,753.00	58,599.26	57.45	57	0.458
G9	Rasch	55,237.36	55,359.36	55,653.97	-	-	-
	2PL	54,611.38	54,851.38	55,430.96	625.98	59	<0.001
	3PL	54,496.82	54,856.82	55,726.18	114.56	60	<0.001
G10	Rasch	68,866.14	68,988.15	69,289.44	-	-	-
	2PL	68,145.38	68,385.39	68,978.10	720.76	59	<0.001
	3PL	67,913.50	68,273.51	69,162.58	231.88	60	<0.001
G11	Rasch	66,987.18	67,109.18	67,408.68	-	-	-
	2PL	66,123.58	66,363.59	66,952.76	863.59	59	<0.001
	3PL	65,964.60	66,324.60	67,208.35	159.00	60	<0.001
G12	Rasch	70,652.76	70,774.76	71,073.77	-	-	-
	2PL	69,520.38	69,760.37	70,348.58	1132.39	59	<0.001
	3PL	69,385.08	69,745.07	70,627.38	135.30	60	<0.001

*Note*. 1PL = Rasch Model; 2PL = Two-Parameter Logistic Model; 3PL = Three-Parameter Logistic Model; −2LL = −2 Log-Likelihood; AIC = Akaike Information Criterion; BIC = Bayesian Information Criterion; LRT = Likelihood Ratio Test.

### Appendix A.2. Anchor Item Screening

To ensure the stability of the linking, we combined DIF indices based on LRT with visual inspection of scatterplots of item difficulty parameters (*b*) between adjacent grades (Figure A1). After the screening process, six items with highly irregular response patterns were removed, and thirteen items showing severe DIF in specific grade pairs were downgraded to non-anchor status. The proportion of anchor items for each grade is presented in Table 3 of the main text.

## Appendix B. Comparison of IRT Linking Methods and Scaled Parameters

**Table A2:** Comparison of Linking Constants across Four IRT Linking Methods.

Path	Haebara		Stocking-Lord		Mean-Mean		Mean-Sigma	
	*A* (*SE*)	*B* (*SE*)	*A* (*SE*)	*B* (*SE*)	*A* (*SE*)	*B* (*SE*)	*A* (*SE*)	*B* (*SE*)
G1 to G7	0.62 (0.05)	−2.95 (0.14)	0.67 (0.05)	−3.07 (0.14)	0.63 (0.05)	−3.37 (0.16)	1.49 (0.29)	−3.75 (0.31)
G2 to G7	0.69 (0.05)	−2.61 (0.11)	0.74 (0.05)	−2.71 (0.12)	0.72 (0.05)	−2.97 (0.14)	1.62 (0.25)	−2.89 (0.21)
G3 to G7	0.67 (0.05)	−2.10 (0.09)	0.71 (0.05)	−2.14 (0.09)	0.75 (0.05)	−2.40 (0.11)	1.07 (0.14)	−2.25 (0.14)
G4 to G7	0.65 (0.04)	−1.56 (0.06)	0.67 (0.04)	−1.62 (0.07)	0.67 (0.04)	−1.83 (0.09)	0.84 (0.10)	−1.61 (0.10)
G5 to G7	0.67 (0.04)	−1.14 (0.05)	0.68 (0.04)	−1.20 (0.06)	0.69 (0.04)	−1.40 (0.08)	0.77 (0.08)	−1.22 (0.09)
G6 to G7	0.67 (0.03)	−0.97 (0.05)	0.72 (0.03)	−1.00 (0.05)	0.76 (0.04)	−1.15 (0.07)	0.71 (0.07)	−1.14 (0.07)
G8 to G7	1.00 (0.05)	0.63 (0.06)	1.04 (0.05)	0.63 (0.06)	1.09 (0.06)	0.64 (0.07)	0.96 (0.07)	0.49 (0.09)
G9 to G7	0.84 (0.05)	1.04 (0.08)	0.87 (0.05)	1.19 (0.08)	0.91 (0.06)	1.35 (0.11)	0.69 (0.09)	0.92 (0.14)
G10 to G7	0.75 (0.06)	0.96 (0.08)	0.81 (0.06)	1.13 (0.08)	0.86 (0.06)	1.32 (0.12)	0.65 (0.11)	0.90 (0.14)
G11 to G7	0.59 (0.06)	1.62 (0.12)	0.62 (0.06)	1.95 (0.13)	0.70 (0.06)	2.56 (0.22)	0.29 (0.06)	1.30 (0.20)
G12 to G7	0.65 (0.07)	1.93 (0.14)	0.70 (0.07)	2.28 (0.15)	0.77 (0.08)	3.00 (0.24)	0.42 (0.10)	1.54 (0.24)

**Table A3:** Descriptive Statistics of Ability and Item Parameters after Equating (Haebara Method).

Grade	Ability Parameters		Item Parameters			
	θ		a		b	
	M	SD	M	SD	M	SD
G1	−2.95	0.57	1.6	0.5	−2.97	2.22
G2	−2.61	0.65	1.65	0.56	−3.21	0.46
G3	−2.1	0.63	1.68	0.6	−2.68	0.48
G4	−1.56	0.61	1.59	0.59	−2.3	0.52
G5	−1.14	0.62	1.61	0.58	−2	0.51
G6	−0.97	0.63	1.47	0.64	−1.47	0.85
G7	0	0.94	0.92	0.41	−0.98	1.15
G8	0.63	0.94	1.04	0.37	−0.61	1.01
G9	1.04	0.78	0.96	0.4	0.09	2.43
G10	0.96	0.7	1.18	0.46	0.35	0.85
G11	1.62	0.55	1.41	0.63	1.29	4.66
G12	1.93	0.61	1.31	0.63	1.75	0.92

*Note*. Item and ability parameters were converted to the Grade 7 reference scale using the Haebara linking constants.

## Appendix C. Comparative Analysis Using Grade-Standardized Raw Scores

To evaluate the empirical performance of the measurement approach adopted in this study, we conducted a comparative analysis by re-estimating the same hierarchical regression models using within-grade standardized raw vocabulary scores (see Table A4).

The results indicated that when using vertically scaled IRT-based latent ability scores (θ), vocabulary ability showed stable, linear, and cross-grade comparable negative associations with all three internalizing symptoms. The interaction term (vocabulary × grade) was statistically significant, with the effect being most pronounced in Grade 8.

However, when within-grade standardized raw scores were used instead, although overall model fit improved slightly, after introducing the interaction term, the explanatory power of Model 3 showed almost no increase (ΔR^2^ ≈ 0). Moreover, some main and interaction effects were in a direction contrary to theoretical expectations. For example, vocabulary scores exhibited significantly positive regression coefficients for all three internalizing symptoms across the junior secondary grades, and in the Grade 9 subsample, vocabulary ability even showed a positive prediction for depression.

This outcome may stem from the inherent limitations of the CTT measurement framework: within-grade standardized scores reflect only a student’s relative position within a specific grade cohort, and cannot distinguish between systematic grade-level differences and true individual ability differences. As a result, regression coefficients may be biased in direction and the model’s explanatory power diminished.

**Table A4:** Summary of Hierarchical Regression Models Using Grade-Standardized Raw Vocabulary Scores.

	Depression			Anxiety			Stress		
Predictor	Model 1	Model 2	Model 3	Model 1	Model 2	Model 3	Model 1	Model 2	Model 3
	B (SE)	B (SE)	B (SE)	B (SE)	B (SE)	B (SE)	B (SE)	B (SE)	B (SE)
** Step 1: Control Variables **									
(Intercept)	4.69 (0.41) ***	3.92 (0.35) ***	3.89 (0.35) ***	4.79 (0.40) ***	4.49 (0.32) ***	4.49 (0.33) ***	5.71 (0.42) ***	5.12 (0.33) ***	5.11 (0.33) ***
Gender	0.31 (0.52)	−0.37 (0.33)	−0.33 (0.33)	0.94 (0.50) ^+^	0.23 (0.30)	0.23 (0.31)	0.90 (0.53) ^+^	0.12 (0.31)	0.14 (0.32)
Only Child	−0.71 (0.52)	0.28 (0.34)	0.30 (0.34)	−0.69 (0.50)	0.29 (0.31)	0.29 (0.31)	−0.82 (0.53)	0.24 (0.32)	0.24 (0.32)
** Step 2: Main Effects **									
Grade (G8 vs. G7)		1.24 (0.41) **	1.24 (0.41) **		0.92 (0.37) *	0.92 (0.37) *		0.97 (0.38) *	0.97 (0.38) *
Grade (G9 vs. G7)		0.82 (0.40) *	0.81 (0.40) *		−0.19 (0.36)	−0.19 (0.37)		0.58 (0.38)	0.58 (0.38)
Vocab. Score (Standardized)		3.90 (0.17) ***	3.47 (0.29) ***		3.91 (0.15) ***	3.85 (0.26) ***		4.17 (0.16) ***	4.04 (0.27) ***
** Step 3: Interaction Effects **									
Vocab. Score × G8			0.49 (0.41)			0.02 (0.37)			0.17 (0.39)
Vocab. Score × G9			0.81 (0.40) *			0.17 (0.37)			0.21 (0.38)
** Model Fit Statistics **									
*R* ^2^	0.01	0.6	0.6	0.01	0.65	0.65	0.01	0.66	0.66
Δ*R*^2^		0.59 ***	0		0.64 ***	0		0.65 ***	0
F	* F * (2, 383) = 1.00	* F * (5, 380) = 114.12 ***	* F * (7, 378) = 82.59 ***	* F * (2, 388) = 2.45 ^+^	* F * (5, 385) = 140.23 ***	* F * (7, 383) = 99.77 ***	* F * (2, 387) = 2.37	* F * (5, 384) = 146.32 ***	* F * (7, 382) = 104.11 ***

*Note*. Coefficients (*B*) are unstandardized estimates with standard errors in parentheses. Gender reference group = male; only-child reference = yes; grade reference = Grade 7. Continuous variables were mean-centered. ^+^ *p* < 0.10. * *p* < 0.05. ** *p* < 0.01. *** *p* < 0.001.
